# Supplementary figures and images for: Global Distribution of mcr Gene Variants in 214K Metagenomic Samples
Source: mSystems. 2022 Mar 28;7(2):e00105-22. doi: 10.1128/msystems.00105-22 (PMC9040840; doi:10.1128/msystems.00105-22)

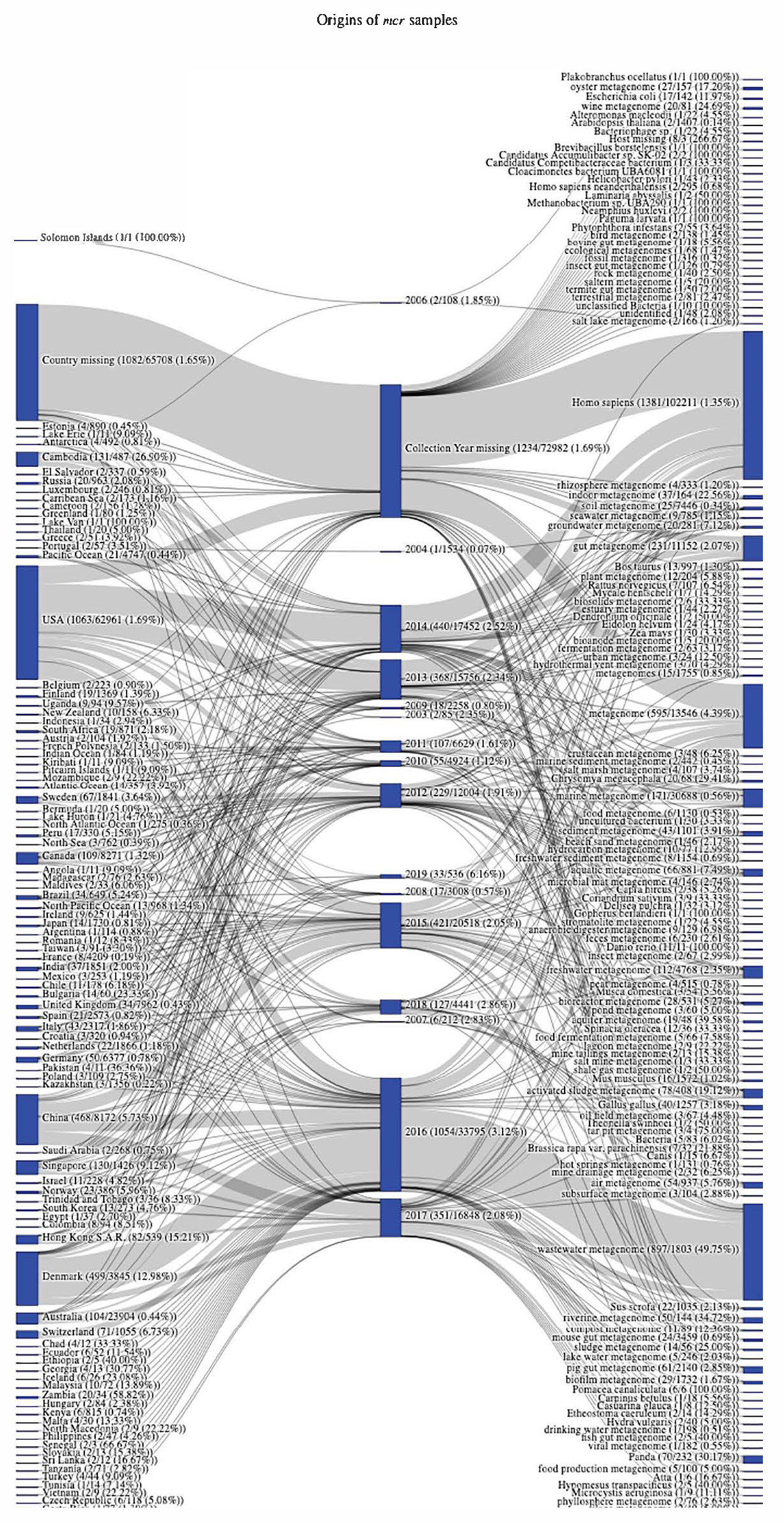

Supplement: FIG S1 [file msystems.00105-22-sf001.tif]

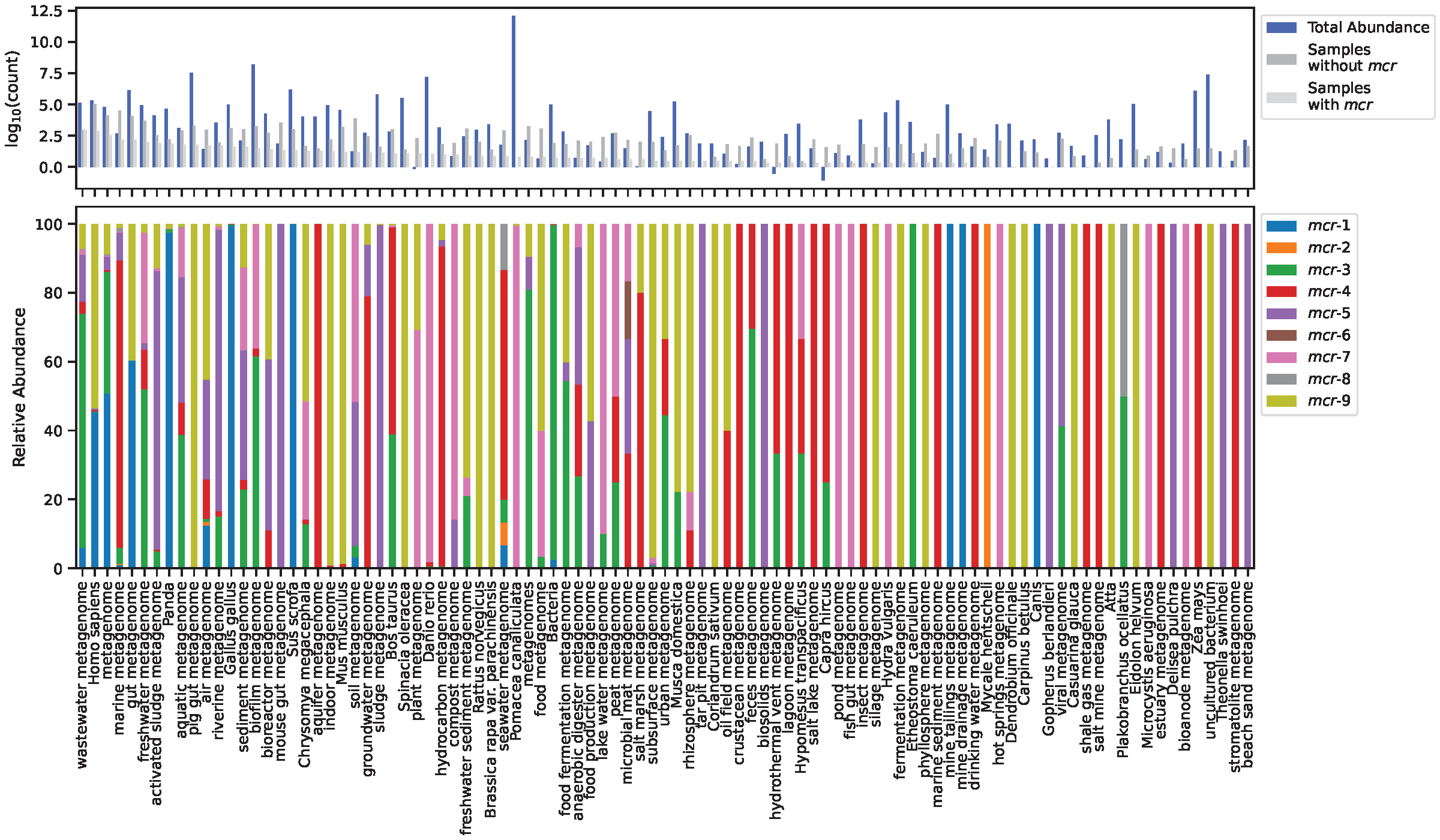

Supplement: FIG S2 [file msystems.00105-22-sf002.tif]

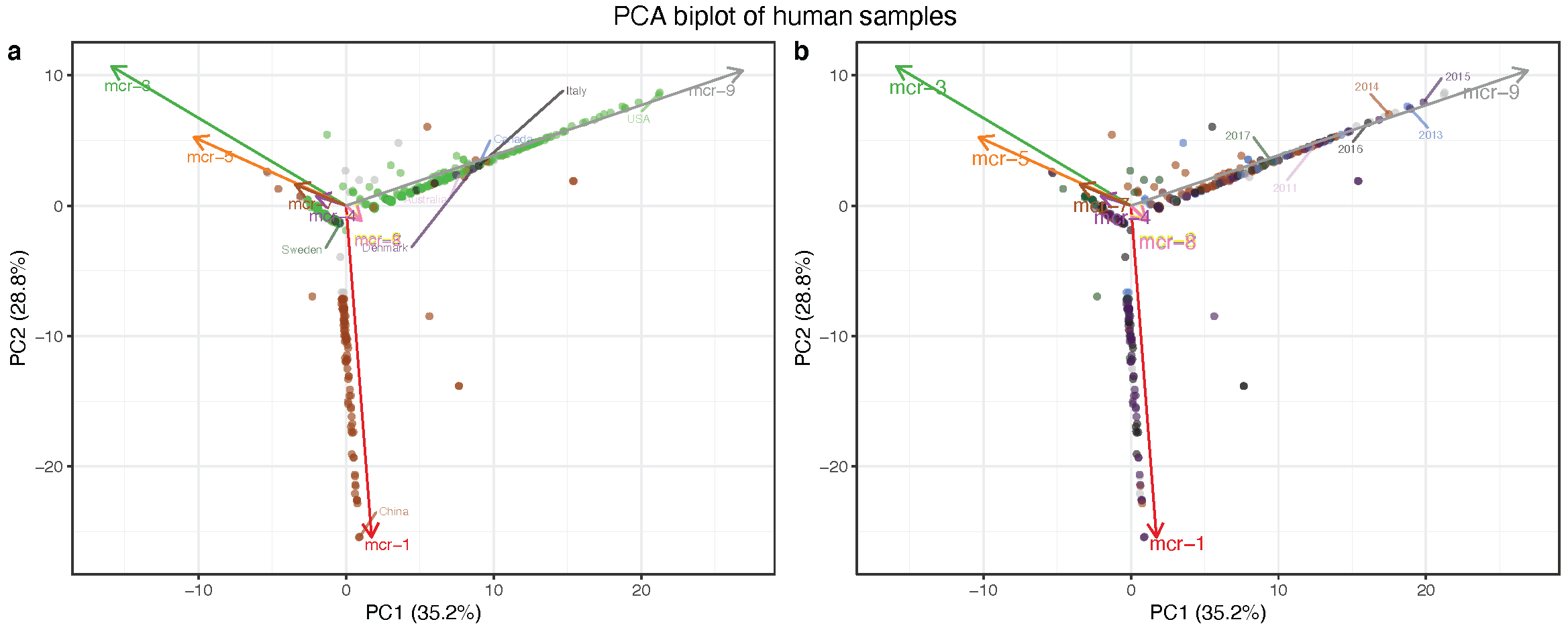

Supplement: FIG S3 [file msystems.00105-22-sf003.tif]

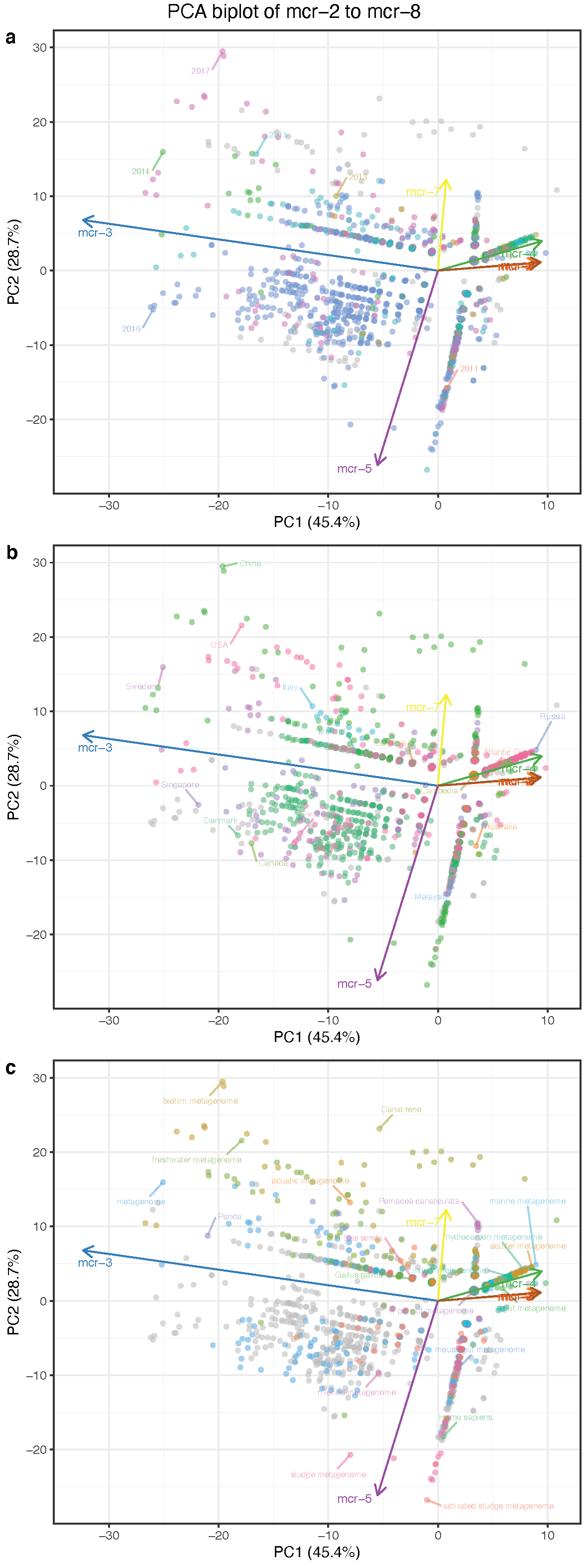

Supplement: FIG S4 [file msystems.00105-22-sf004.tif]

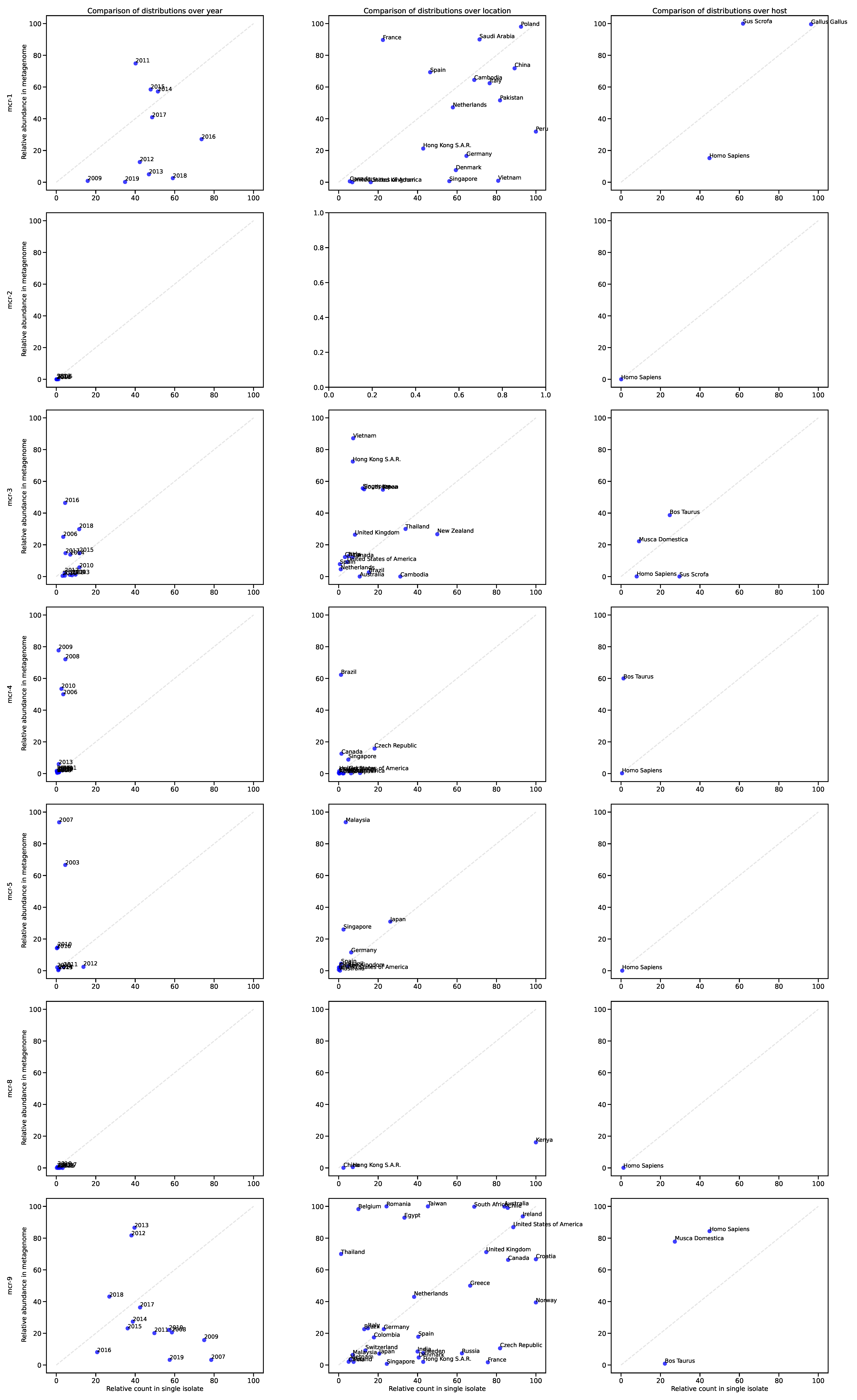

Supplement: FIG S5 [file msystems.00105-22-sf005.tif]

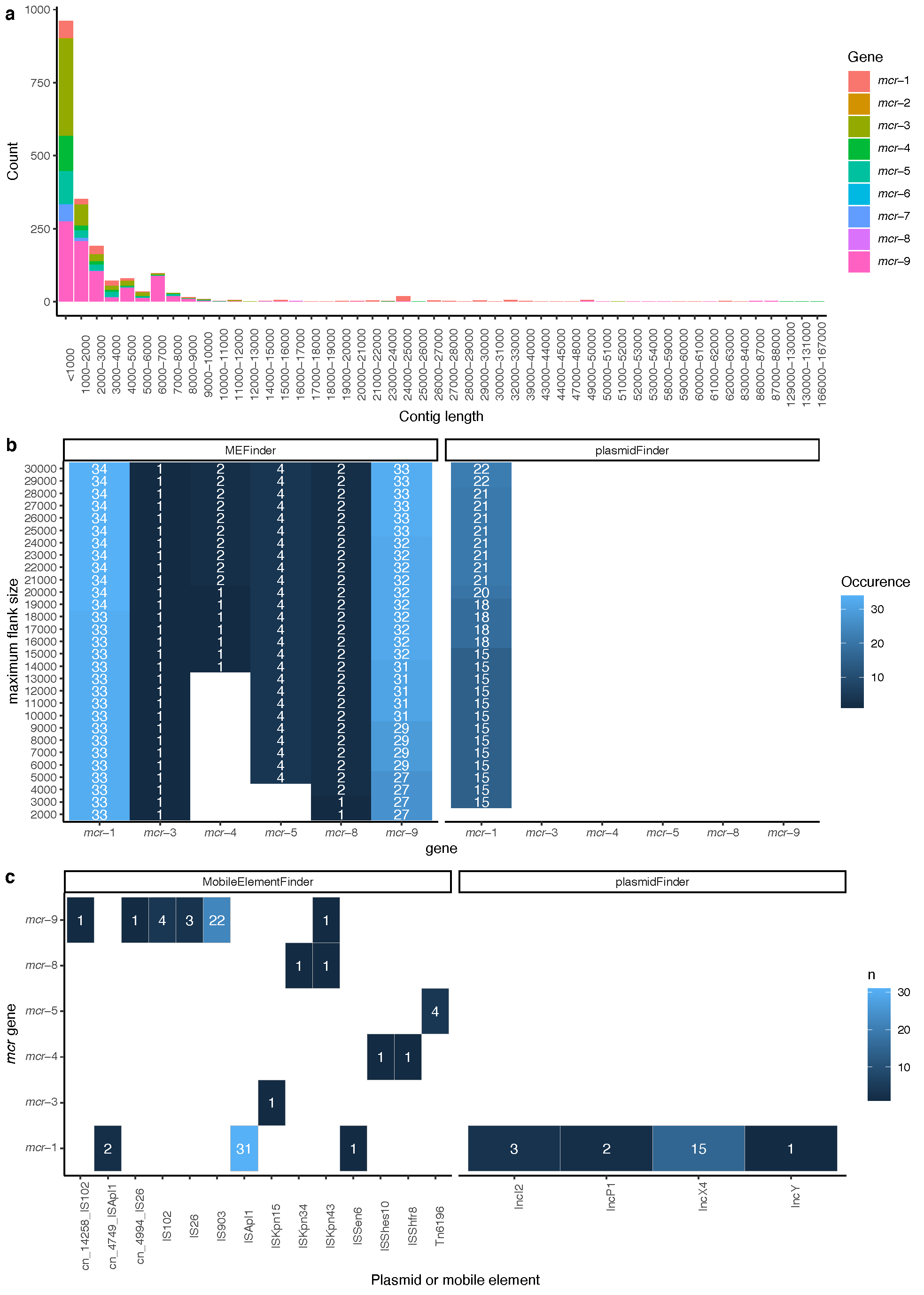

Supplement: FIG S6 [file msystems.00105-22-sf006.tif]

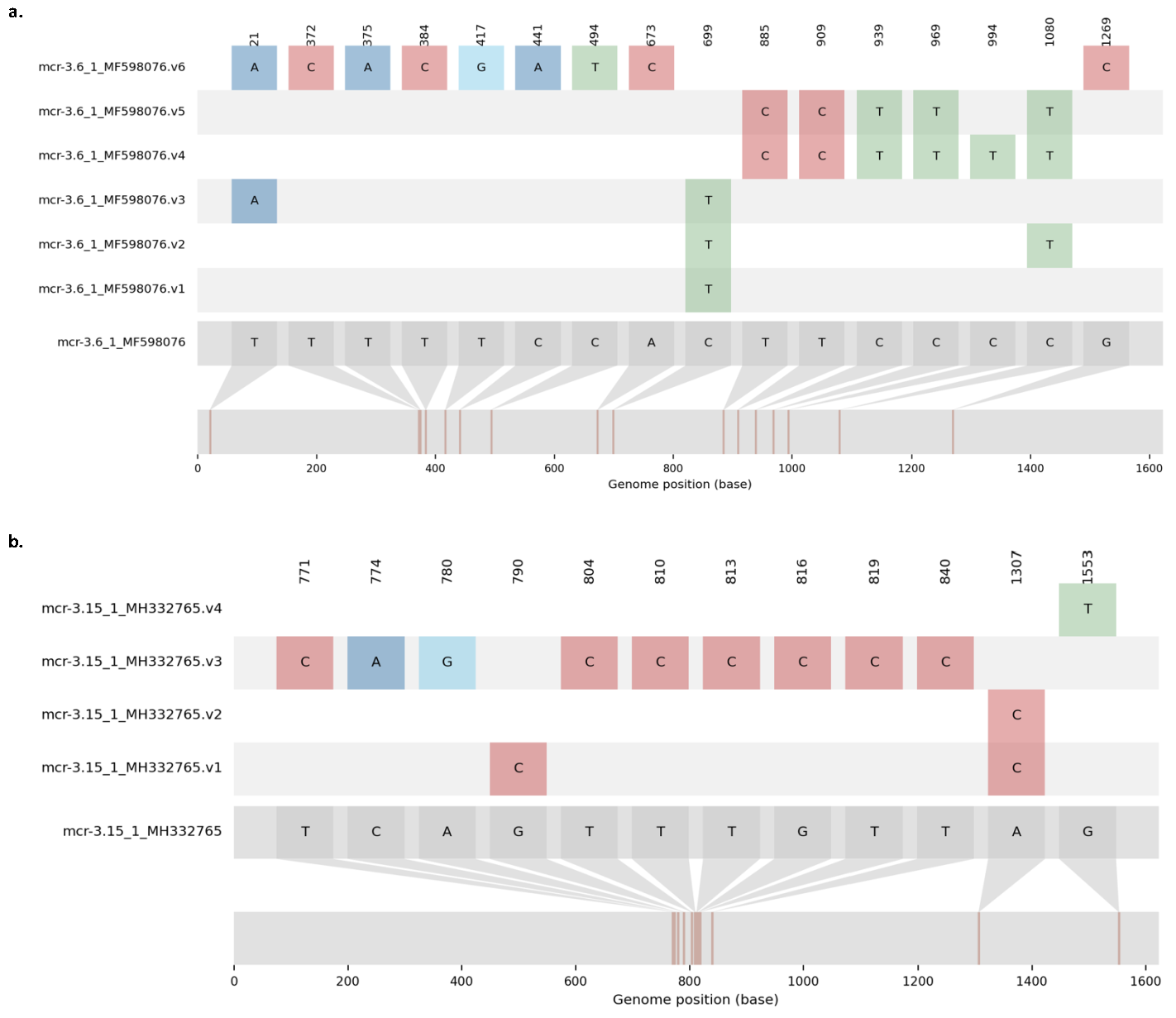

Supplement: FIG S7 [file msystems.00105-22-sf007.tif]

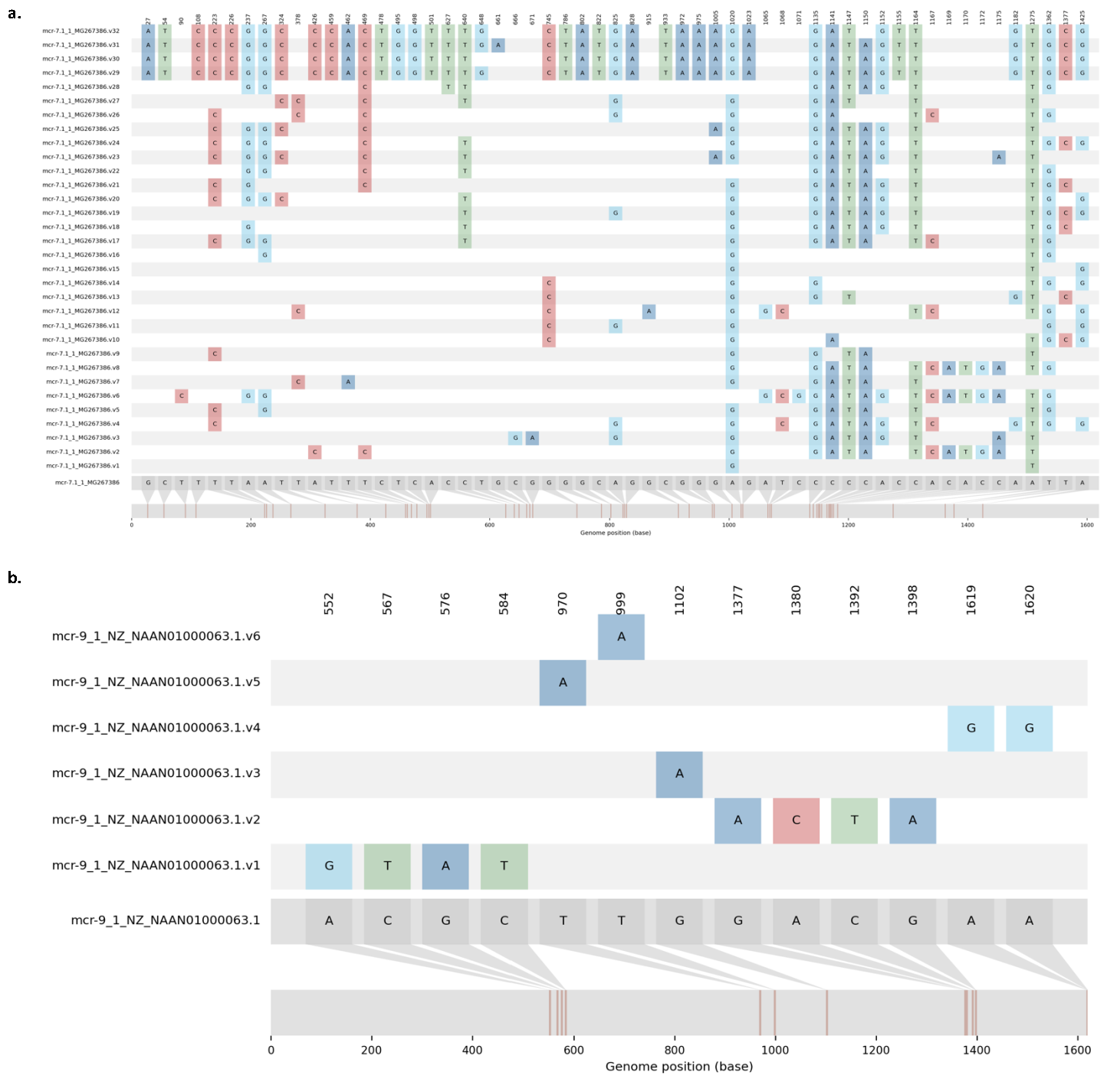

Supplement: FIG S8 [file msystems.00105-22-sf008.tif]
