# Supplementary material for: Global Distribution of mcr Gene Variants in 214K Metagenomic Samples
Source: mSystems. 2022 Mar 28;7(2):e00105-22. doi: 10.1128/msystems.00105-22 (PMC9040840; doi:10.1128/msystems.00105-22)
Supplement: TABLE S1 [file msystems.00105-22-st001.docx]

|  | *mcr-1* | *mcr-2* | *mcr-3* | *mcr-4* | *mcr-5* | *mcr-6* | *mcr-7* | *mcr-8* | *mcr-9* |
| --- | --- | --- | --- | --- | --- | --- | --- | --- | --- |
| Isolate frequency (%) | 51.08 | 0.25 | 6.83 | 0.74 | 1.26 | 0.00 | 0.00 | 1.18 | 40.38 |
