# Supplementary material for: Global Distribution of mcr Gene Variants in 214K Metagenomic Samples
Source: mSystems. 2022 Mar 28;7(2):e00105-22. doi: 10.1128/msystems.00105-22 (PMC9040840; doi:10.1128/msystems.00105-22)
Supplement: TABLE S2 [file msystems.00105-22-st002.docx]

|  | ***mcr*-1.1.v2** | ***mcr*-3.6.v1** | ***mcr*-3.6.v3** | ***mcr*-3.15.v2** | ***mcr*-4.3.v3** | ***mcr*-4.3.v4** | ***mcr*-4.3.v5** | ***mcr*-5.1.v1** | ***mcr*-8_1.v1** | ***mcr*-9_1.v4** |
| --- | --- | --- | --- | --- | --- | --- | --- | --- | --- | --- |
| Total metagenomic hits | 1 | 14 | 1 | 9 | 8 | 1 | 1 | 1 | 1 | 139 |
| Total BLAST hits | 17 | 3 | 4 | 3 | 25 | 1 | 1 | 5 | 20 | 100 |
| **Species** |  |  |  |  |  |  |  |  |  |  |
| Acinetobacter baumannii |  |  |  |  | 5 |  |  |  |  |  |
| Acinetobacter nosocomialis |  |  |  |  | 1 |  |  |  |  |  |
| Aeromonas hydrophila |  |  | 1 | 2 |  |  |  |  |  |  |
| Aeromonas media |  |  | 3 |  |  |  |  |  |  |  |
| Aeromonas sanarellii |  | 1 |  |  |  |  |  |  |  |  |
| Aeromonas sp. 2692-1 |  |  |  | 1 |  |  |  |  |  |  |
| Aeromonas veronii |  | 2 |  |  |  |  |  |  |  |  |
| Citrobacter freundii |  |  |  |  |  |  |  |  |  | 4 |
| Citrobacter sp. |  |  |  |  |  |  |  |  |  | 1 |
| Citrobacter telavivensis |  |  |  |  |  |  |  |  |  | 1 |
| Enterobacter asburiae |  |  |  |  |  |  |  |  |  | 3 |
| Enterobacter cancerogenus |  |  |  |  |  |  |  |  |  | 1 |
| Enterobacter cloacae |  |  |  |  | 3 |  |  |  |  | 3 |
| Enterobacter cloacae complex sp. |  |  |  |  |  |  |  |  |  | 2 |
| Enterobacter hormaechei |  |  |  |  |  |  |  |  |  | 31 |
| Enterobacter hormaechei subsp. xiangfangensis |  |  |  |  |  |  |  |  |  | 1 |
| Enterobacter kobei |  |  |  |  | 1 |  |  |  |  | 2 |
| Enterobacter roggenkampii |  |  |  |  |  |  |  |  |  | 1 |
| Enterobacter sp. BWH 37 |  |  |  |  |  |  |  |  |  | 1 |
| Enterobacter sp. MGH 14 |  |  |  |  |  |  |  |  |  | 1 |
| Enterobacter sp. T2 |  |  |  |  |  |  |  |  |  | 1 |
| Escherichia coli | 12 |  |  |  |  |  |  | 1 |  | 9 |
| Expression vector pUC57-Kan-mcr-9 |  |  |  |  |  |  |  |  |  | 1 |
| Hafnia paralvei |  |  |  |  |  |  |  |  |  | 1 |
| Klebsiella grimontii |  |  |  |  |  |  |  |  |  | 1 |
| Klebsiella oxytoca |  |  |  |  |  |  |  |  |  | 1 |
| Klebsiella pneumoniae | 5 |  |  |  |  |  |  |  | 18 | 2 |
| Klebsiella quasipneumoniae |  |  |  |  |  |  |  |  | 1 |  |
| Leclercia adecarboxylata |  |  |  |  | 1 |  |  |  |  | 2 |
| Leclercia sp. |  |  |  |  |  |  |  |  |  | 2 |
| Lelliottia amnigena |  |  |  |  | 1 |  |  |  |  |  |
| mixed culture bacterium |  |  |  |  | 1 |  |  |  |  |  |
| Salmonella enterica |  |  |  |  |  |  |  |  |  | 2 |
| Salmonella enterica subsp. diarizonae serovar b,50:-:- |  |  |  |  |  |  |  |  |  | 1 |
| Salmonella enterica subsp. enterica serovar 4,[5],12:i:- |  |  |  |  |  |  |  |  |  | 3 |
| Salmonella enterica subsp. enterica serovar 4,5,12:i- |  |  |  |  |  |  |  |  |  | 3 |
| Salmonella enterica subsp. enterica serovar Albany |  |  |  |  |  |  |  |  |  | 2 |
| Salmonella enterica subsp. enterica serovar Brandenburg |  |  |  |  |  |  |  |  |  | 1 |
| Salmonella enterica subsp. enterica serovar Dessau |  |  |  |  |  |  |  |  |  | 2 |
| Salmonella enterica subsp. enterica serovar Heidelberg |  |  |  |  |  |  |  |  |  | 4 |
| Salmonella enterica subsp. enterica serovar Johannesburg |  |  |  |  |  |  |  |  |  | 1 |
| Salmonella enterica subsp. enterica serovar Newport |  |  |  |  |  |  |  |  |  | 1 |
| Salmonella enterica subsp. enterica serovar Saintpaul |  |  |  |  |  |  |  |  |  | 4 |
| Salmonella enterica subsp. enterica serovar Schwarzengrund |  |  |  |  |  |  |  |  |  | 2 |
| Salmonella enterica subsp. enterica serovar Westhampton |  |  |  |  |  |  |  |  |  | 1 |
| Serratia marcescens |  |  |  |  |  |  |  |  |  | 1 |
| Shewanella frigidimarina NCIMB 400 |  |  |  |  | 1 |  |  |  |  |  |
| uncultured bacterium |  |  |  |  | 9 | 1 | 1 | 4 | 1 |  |
| Acinetobacter baumannii |  |  |  |  | 5 |  |  |  |  |  |
| Acinetobacter nosocomialis |  |  |  |  | 1 |  |  |  |  |  |
